# The complete chloroplast genome of Mediterranean shrub *Teucrium fruticans* L. (Lamiaceae; Subfam. Lamioideae)

**DOI:** 10.1080/23802359.2022.2090298

**Published:** 2022-07-07

**Authors:** Shoufu Gong

**Affiliations:** School of Horticulture, Xinyang Agriculture and Forestry University, Xinyang, China

**Keywords:** *Teucrium fruticans*, chloroplast genome, Mediterranean region, ornamental plants

## Abstract

*Teucrium fruticans* L. is a shrub in Lamiaceae that is native to Mediterranean countries where it is used in medicine ornamental gardens. Here, we report the complete chloroplast genome of *T. fruticans* which is 150,808 bp in length, with a pair of inverted repeat regions (IRs) (25,597 bp) separated by a large single-copy area (LSC) (82,634 bp) and a small single-copy area (SSC) (16,912 bp). The completed chloroplast genome of *T. fruticans* comprises 130 unique genes, including 86 protein-coding genes, 36 tRNA genes, and eight rRNA genes. The results of the phylogenetic analysis significantly supported the grouping of *T. fruticans* with nine *Teucrium* species. The complete chloroplast genome of *T. fruticans* can provide a powerful tool to accelerate breeding, biotechnological and phylogenetic study.

The genus *Teucrium* consists of approximately 250 species; most of which have ornamental and medicinal uses, including *Teucrium fruticans* L. (1753) (Lopez et al. 2006; Frabetti et al. 2009; Salmaki et al. 2016; Gagliano Candela et al. 2021). Owing to its brilliant blue flowers and evergreen foliage with contrasting colors, it is widely planted in ornamental gardens (Acquaviva et al. 2018; Khan et al. 2019). Additionally, this species is used in the traditional folk medicine of Central Italy as a depurative and diuretic (Gagliano Candela et al. 2021). Other studies illustrate that this species contains abundant functional metabolites, such as flavonoids, fatty acid esters, and essential oils (Fontana et al. 1999; Flamini et al. 2001; Kisiel et al. 2014). In the present study, we assembled and annotated the complete cp genome of *T. fruticans*, and analyzed its sequence in a phylogenetic context with other publicly available cp genomes. This is an essential genomic resource for future molecular studies of this species as well as the evolutionary history of Lamiaceae.

Fresh leaves of *T. fruticans* were collected from Xinyang Agriculture and Forestry University (Xinyang, China; 114°13′E, 32°17′N) and frozen in liquid nitrogen before DNA extraction. Voucher specimens were deposited in the herbarium of Xinyang Agriculture and Forestry University (Mr. Gong, 2000230016@xyafu.edu.cn) under the voucher code XTF22003. Total genomic DNA was extracted using the modified CTAB method (Doyle and Doyle 1987). The TruSeq™ DNA sample Prep Kit was used to construct a ∼500 bp library that was sequenced on an Illumina NovaSeq6000 platform for 250 cycles paired end. Raw reads were edited through in-house Perl scripts by removing reads containing adapter or poly-N and low-quality reads, and then clean reads were de novo assembled by NOVOPlasty (Dierckxsens et al. 2017). The assembled chloroplast genome of *T. fruticans* was annotated using PGA (Qu et al. 2019) and GeSeq (Tillich et al. 2017). For each gene, start and stop codons, and boundaries between introns and exons were manually corrected. The annotated cp genome was submitted to GenBank (OL960707) and the raw sequencing data were deposited in SRA (SRA no. PRJNA775852).

The length of the complete chloroplast genome of *T. fruticans* was 150,808 bp with a total GC content of 38.0%. The complete chloroplast genome of *T. fruticans* had a typical quadripartite construction, which contains two inverted repeat regions (IRa and IRb) of 25,597 bp that was segregated by a large single-copy (LSC, 82,634 bp) and a small single-copy (SSC 16,912 bp). The GC content for the LSC and SSC was 35.76% and 31.60%, respectively. The complete chloroplast genome of *T. fruticans* comprised 130 unique genes, including 86 protein-coding genes, 36 tRNA genes, and eight rRNA genes. Introns are present in 23 of the annotated genes.

To confirm the phylogenetic position of *T. fruticans*, the complete chloroplast genomes of 32 Lamiaceae plant species (including nine *Teucrium* species) were downloaded from the NCBI GenBank database. The sequences were aligned using the MAFFT version 7.429 software (Katoh and Standley 2013), and then the maximum-likelihood (ML) tree (Figure 1) was constructed using IQ-TREE with 1000 bootstrap replicates (Nguyen et al. 2015). In the phylogenetic reconstruction, *T. fruticans* formed a clade with the other *Teucrium* species in which it is the earliest branching lineage (Figure 1). This *T. fruticans* chloroplast genome will provide vital genomic data for future studies of horticultural development, population genetics, and evolution in Lamiaceae.

**Figure 1.**
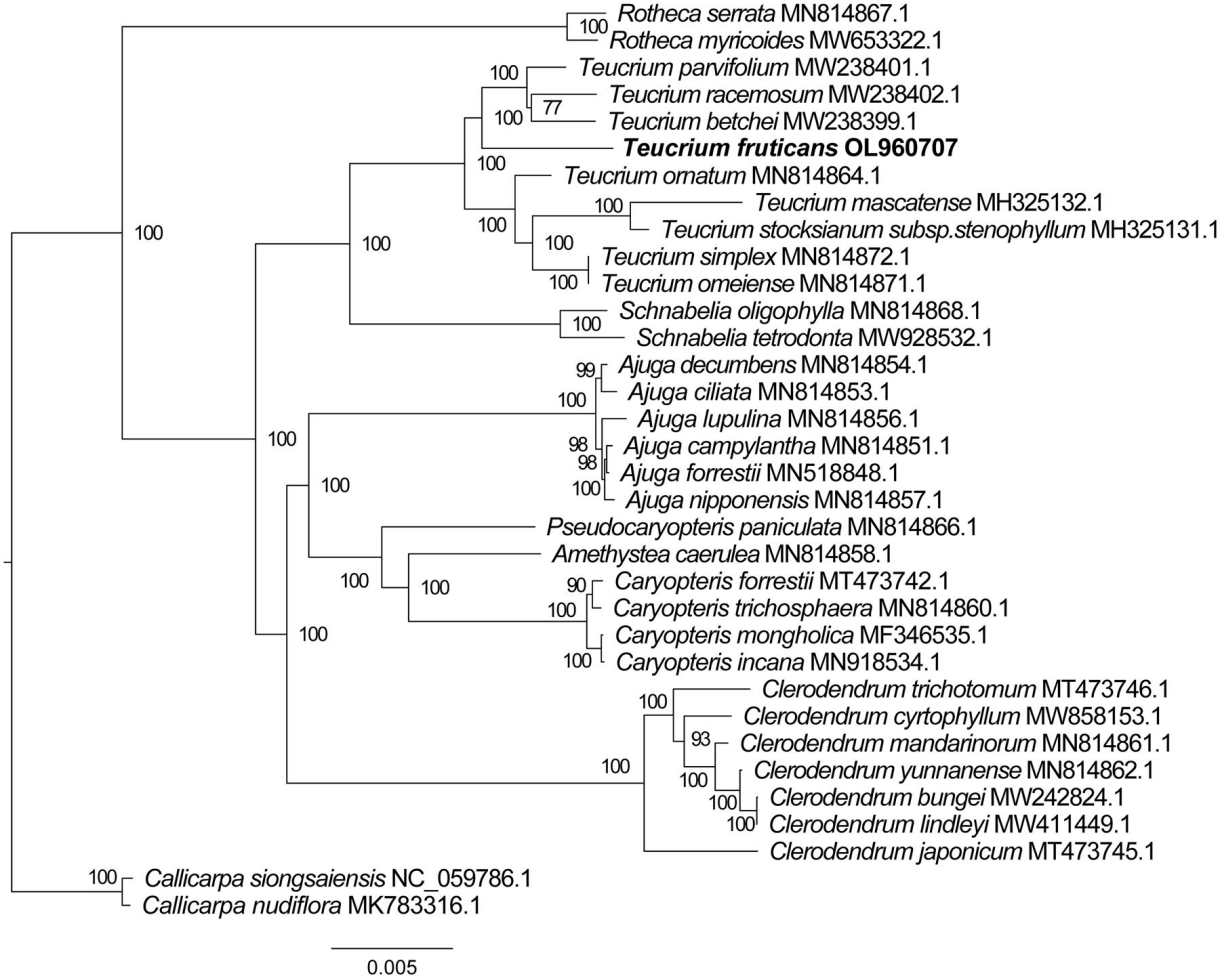
The maximum-likelihood phylogenetic tree showed the relationship between *T. fruticans* and other 31species within the Lamiaceae family, using complete chloroplast genomes. The numbers above the lines represent ML bootstrap values. The scale bar indicates evolutionary distance in substitutions per site.

## Data Availability

The contact person for the specimen is Shoufu Gong (2000230016@xyafu.edu.cn). The genome sequence data that support the findings of this study are openly available in GenBank of NCBI at https://www.ncbi.nlm.nih.gov/ under the accession no. OL960707. The associated BioProject, SRA, and Bio-Sample numbers are PRJNA775852, SRX12809278, and SAMN22630214, respectively.
